# Can a Hybrid Line Break a Selection Limit on Behavioral Evolution in Mice?

**DOI:** 10.1007/s10519-024-10209-7

**Published:** 2024-12-05

**Authors:** Layla Hiramatsu, Vincent Careau, Theodore Garland

**Affiliations:** 1 Department of Evolution, Ecology, and Organismal Biology, University of California, Riverside, CA USA; 2 Present Address: Department of Biology, University of Ottawa, Ottawa, ON Canada

**Keywords:** Artificial selection, Genetic architecture, Heterosis, Hybrid, Voluntary exercise, Wheel running

## Abstract

Artificial selection yielded four replicate high runner (HR) lines of mice that reached apparent selection limits (~ threefold increase in wheel revolutions per day vs. four control lines), despite maintenance of additive genetic variance. After 68 generations, we used animal models to test for changes in additive-genetic variances and covariance of the two measured components (average speed and duration) of running distance. We also attempted to break the selection limit by crossing two HR lines, then continuing directional selection on this hybrid line and on the two parental lines for nine generations. The genetic correlation between speed and duration was positive in the base population, but evolved to be negative in the two parental HR lines. Although heritability for both speed and duration (but not distance) increased in the hybrid line, their genetic correlation remained negative. Hybrid F_1_ mice from generation 68 parents showed heterosis for running distance, which was lost in subsequent generations, and the hybrid line did not exceed the limit. Both male and female hybrids ran faster than parental lines for most generations, but running duration was intermediate or reduced, reflecting their negative genetic correlation. The evolved genetic trade-off between speed and duration may explain the inability for the hybrid line to break the selection limit for distance run, despite renewed additive genetic variance for at least one of its component traits.

**Supplementary Information:**

The online version contains supplementary material available at 10.1007/s10519-024-10209-7.

## Introduction

Limits to selection (plateaus) are common in selection experiments and can result from various causes (Falconer and MacKay 1996; Garland and Rose 2009; Careau et al. 2013). Perhaps the most intuitive potential cause of a selection limit is simply exhaustion of additive genetic variance (i.e., the narrow-sense heritability is reduced to zero). In a population under selection, both the selection regime and genetic drift would cause fixation of alleles, with selection fixing beneficial alleles and genetic drift fixing alleles without regard of their relevance for the selection regime (Falconer and MacKay 1996). Fixation of alleles is especially likely under strong directional selection and/or with small population sizes (Weber 1996), as is the case for most artificial selection experiments with rodents (e.g., Meyer and Hill 1991; Beniwal et al. 1992; Heath et al. 1995).

Another possible cause of selection limits is counterposing natural selection (e.g., selected lines suffer a dramatic decrease in fertility), which can be viewed as a consequence of adverse pleiotropic effects of alleles under selection (Barton and Turelli 1989; Hill and Mbaga 1998). For example, two selection experiments for body mass in mice resulted in decreased fertility and postnatal survival in lines at or near plateaus (Falconer 1955; Roberts 1966). Of particular relevance for the present study, selective breeding for high home-cage locomotor activity in mice led to decreased reproductive success (Majdak et al. 2014). Inbreeding depression in small populations may also decrease reproductive success and other aspects of Darwinian fitness (Falconer and MacKay 1996; Birchler et al. 2006; Charlesworth and Willis 2009; Pemberton et al. 2016).

One recent example of a selection experiment that reached apparent limits involves selection for high voluntary wheel-running behavior in laboratory mice (*Mus domesticus*; Hsd:ICR strain). Replicated directional selection for this trait produced four High Runner (HR) lines of mice that run ~3 times as much as four non-selected control (C) lines at selection limits (Swallow et al. 1998; Careau et al. 2013). Although all replicate lines show approximately the same increase in total wheel revolutions per day, they differ in the component traits of average running speed and running duration per day (Garland et al. 2011), and an apparent trade-off between these components of running behavior has emerged (see Fig. 3 and Supplemental Fig. 1 in Garland et al. 2011). Considering all four of the replicates, the HR lines have evolved a variety of other consistent differences as compared with the four control lines, including reduced body mass and length (Swallow et al. 1999), higher endurance (Meek et al. 2009) and maximal aerobic capacity (Rezende et al. 2006; Schwartz et al. 2023) during forced exercise on a treadmill, and larger hearts and brains (Kolb et al. 2013; Copes et al. 2015). The HR lines also show an altered brain reward system, including in the dopamine (Rhodes and Garland 2003), serotonin (Claghorn et al. 2016), and endocannabinoid pathways (Thompson et al. 2017). Analyses of whole-genome sequences and gene expression data have begun to identify divergent chromosomal regions and potentially causal genetic loci (Saul et al. 2017; Hillis et al. 2020, 2024; Nguyen et al. 2020), as well as “multiple solutions” at the genomic level (Hillis and Garland 2023).

**Fig. 1:**
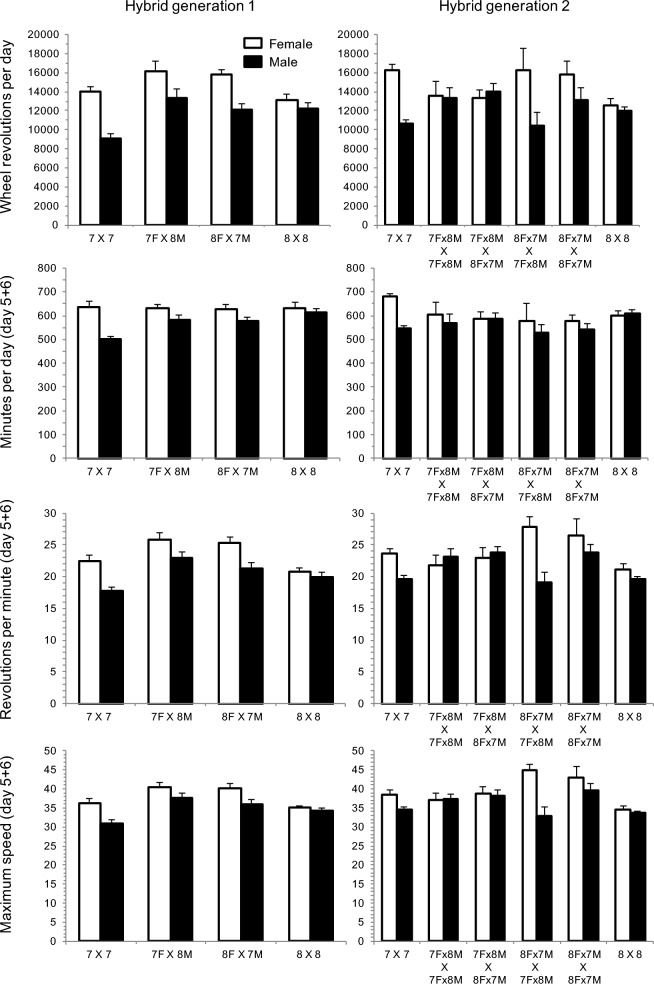
Wheel running and component traits for hybrid generations 1 (corresponding to generation 69) and 2 (measured as mean of days 5 + 6 of a 6-day exposure to wheels). Values are least-squares means ± standard errors from analysis of covariance models in SAS Procedure Mixed, performed separately for the two sexes. 7 × 7 and 8 × 8 denote purebred mice from HR lines 7 and 8. Left panels are mice from hybrid generation 1 and show purebred mice and reciprocal hybrid crosses. Right panels are mice from hybrid generation 2 and show purebred mice and 4-way crosses of the reciprocal hybrid mice. For example, 7Fx8M × 8Fx7M denotes offspring from crosses of females from the F_1_ reciprocal 7F × 8 M with males from the F_1_ reciprocal 8F × 7 M. No parent-of-origin effect was apparent for wheel-running distance or component traits in the reciprocal hybrids (p > 0.05 for contrast between 7F × 8 M vs. 8F × 7 M in both sexes). Grand-parental effects were apparent for females whose mothers were from the F_1_ cross 8F × 7 M compared with females whose mothers were from the F_1_ cross 7F × 8 M, with significantly increased running speed (*p* = 0.0155), but no difference in total wheel-running distance (*p* = 0.1376) or duration (*p* = 0.7396). Males whose mothers were the F_1_ cross 8F × 7 M and fathers were the F_1_ cross 7F × 8 M tended to have reduced running distance (*p* = 0.0580) and had significantly reduced speed (*p* = 0.0121) compared with males from the other three F_2_ crosses

The HR lines reached selection limits between generations 17–25, differing slightly based on replicate line and sex within line (Careau et al. 2013). The limit experienced in the HR lines was apparently not caused by depleted additive genetic variance for daily wheel-running distance, counterpoising natural selection, or changes in the additive-genetic variance–covariance matrix for running across the six days of the trial used to select breeders each generation (Girard et al. 2002; Careau et al. 2013; Careau et al., 2015; Khan et al. 2024). The purpose of the present study was to: (1) analyze the genetic correlation between the measured components of daily wheel-running distance (average speed and duration) in the base population and in current generations; and (2) attempt to break the selection limit experienced by the HR lines by continued selection in a hybrid line.

A hybrid line of two replicate selected populations may allow breaking of a selection limit by recouping additive-genetic variance for the trait under selection. Especially in small populations, random genetic drift would result in the loss of some favorable alleles before selection could “recruit” them, and these lost alleles would likely differ among replicate populations (Bell 2008, p. 213). Moreover, as mentioned previously, random genetic drift can potentially fix alleles with neutral or detrimental effects, and will, on average, cause populations to diverge genetically. Thus, in principle, a cross of two replicate selected lines will inherit favored genes from both, replenishing alleles lost by drift in one or the other replicate lines, and hence can respond to renewed directional selection. Empirical evidence that this approach can indeed be used to break selection limits can be found in a selection experiment on nest-building behavior in mice. Replicate lines bred for high and low thermoregulatory nest-building reached selection limits at around generation 20 (Lynch 1994). Replicate hybrid lines were created at generation 46 for both the high and low selected lines, and all 4 hybrid lines broke selection limits after ~ 8–10 generations of renewed selection (Bult and Lynch 2000). Here, we implemented the paradigm outlined by Bult and Lynch (2000) in an attempt to break the selection limit reached in the HR mice.

## Materials and methods

### Original selected lines

We used laboratory mice (*Mus musculus*) that had undergone 68 generations of directional selection for high levels of voluntary wheel running (Swallow et al. 1998; Careau et al. 2013). The base population for this long-term selection experiment was 224 unrelated mice from the genetically variable, outbred Hsd:ICR strain (Harlan-Sprague–Dawley, Institute of Cancer Research). After 2 generations of random mating, we established 4 high-runner (HR) and 4 non-selected control (C) lines. At all times throughout the experiment, mice were kept with a 12:12 light–dark cycle at 20–24 degrees Celsius, and food and water were provided ad libitum. As young adults (~ 6–9 weeks of age), all mice were placed in new, individual home cages with access to wheels for 6 days to measure their voluntary wheel running. The wheels were 1.12 m in circumference and attached externally to the home cage, accessed via a tunnel (see Fig. S1 in Kelly et al. 2017). HR lines were bred based on their total wheel revolutions on days 5 and 6. Ten pairs per line were maintained using within-family selection (following Lynch 1994; Bult and Lynch 2000), so that all families were represented in each generation and inbreeding was minimized (N_e_ ~35 per line). That is, we selected the highest-running female and male from each family and mated them to the highest-running male or female from other families. We avoided pairing of siblings. Only first litters were used. The same testing and breeding protocols were followed in the current hybrid line experiment. Note that within-family selection, which was also used by Lynch (Lynch 1994; Bult and Lynch 2000) and Majdak et al. (2014), may result in a lower rate of response to selection, but should also result in a greater total divergence before selection limits are reached. This is in part because it doubles the effective population size and so reduces the rate of inbreeding (Falconer and Mackay 1996; Montaldo and Castillo-Juárez 2017).

A sensor attached to each wheel counted every rotation, and a custom computer program recorded the number of rotations in 1-min intervals for 23 h/day. Once a day before starting the next test, we checked every cage for the health of the mouse and that the wheels were turning freely. After 6 days, we took mice out of the wheel cages and placed them back in standard housing cages in same-sex groups of 4. Daily metrics of wheel running were: total revolutions, number of 1-min intervals with any revolutions, average speed (total revolutions divided by number of 1-min intervals active), and maximum speed (the highest number of revolutions in any 1-min interval). We weighed all mice before placing them in the wheel cages on day 1 and when we took them out of the wheel cages at the end of day 6.

### Hybrid line

In the selection experiment, assignment of lines to the Control (lab designated lines 1, 2, 4, 5) and HR treatments (lab designated lines 3, 6, 7, 8) was done at random. Previously, Hannon et al. (2011) observed heterosis for wheel running in male (but not female) hybrids of HR lines 7 and 8, suggesting that continued selection on the hybrid line would have the potential to break the selection limit that exists in the HR lines, although possibly only for males. Therefore, we crossed HR lines 7 and 8 and continued directional selection on this hybrid line, and on both parental lines, for nine additional generations (following Bult and Lynch 2000). Owing to financial and logistical constraints, we were only able to create one hybrid line, which involved maintaining and testing ~100 additional mice per generation. As in Hannon et al. (2011), we used HR replicate lines 7 and 8 due to the absence of the mini-muscle allele (fixed in HR line 3 and polymorphic in HR line 6; (Kelly et al. 2013)), which affects many traits, including wheel running and organ masses (e.g., see Garland et al. 2002; Hannon et al. 2008; Hillis and Garland 2023; Khan et al. 2024). Importantly, HR lines 7 and 8 have been found to differ for a variety of traits in previous generations, including body mass, daily run distance, average and maximum wheel-running speed, behavior in an open-field and in an elevated plus-maze test, locomotor play behavior, and the masses of the heart ventricle, spleen, soleus, plantaris, triceps surae, and lower forelimb muscles (all organs analyzed with body mass as a covariate), with some of the differences being sex-specific (McGillivray et al. 2009; Dlugosz et al. 2009; Jonas et al. 2010; Hannon et al. 2011; Whitehead et al. 2023).

At generation 68, in addition to breeding the replicate HR lines as usual, we bred a subset of females and males from lines 7 and 8 to create two reciprocal hybrid crosses (7 female × 8 male and 8 female × 7 male). These two reciprocal crosses were combined to create what we call the F1 that became hybrid line 9.

In creating the next generation (F_2_) of the hybrid line, we imposed selection to the extent possible within the confines of implementing a factorial breeding design to maximize allele mixing and retain the ability to test for grand-parental effects. Specifically, we bred females from one reciprocal cross to males from the same and different crosses: i.e., females from the F_1_ reciprocal 7F × 8 M were bred to males from the F_1_ reciprocal 7F × 8 M or males from the F_1_ reciprocal 8F × 7 M, and the same for females from the F_1_ reciprocal 8F × 7 M. In subsequent generations (F_3+_) of the hybrid line, we combined these crosses as one pool of breeders. We continued selection in the following generations for the parental and hybrid lines, following the usual selection protocol with within-family selection, for a total of 10 generations of the hybrid line. Supplemental Figure S1 depicts the experimental timeline.

### Comparison of lines and line crosses

Analyses were performed separately by sex unless otherwise noted, because of many known differences between sexes (Garland et al. 2011; Hannon et al. 2011). For each generation, we tested whether the hybrid line had diverged significantly from the parental lines using analysis of covariance (ANCOVA) in SAS Procedure Mixed (version 23; SAS Institute, Cary, NC, USA). Analyses of body mass used age as a covariate. Analyses of wheel-running traits used age and wheel freeness as covariates. Wheel freeness was tested for each wheel by accelerating the wheel to a constant velocity and counting revolutions until the wheel stopped on its own (Copes et al. 2015). For analysis, the square-root of wheel freeness was used to obtain a more homogenous spread of values. We tested for the difference between the hybrid line and parental lines using three separate a priori contrasts: hybrid line 9 vs. parental HR line 7, hybrid line 9 vs. parental HR line 8, and hybrid line 9 vs. the average of parental HR lines 7 and 8. We used additional contrasts for F_1_ reciprocal crosses: hybrids created from line 7 females crossed with line 8 males vs. hybrids created from line 8 females crossed with line 7 males. We also used additional contrasts for F_2_ reciprocal crosses.

The hybrid line exhibited greater variance than parental lines, so we considered 6 different models with (1) a single estimate for residual variance, (2) a single estimate for residual variance and a single estimate for variance among families (as a nested random effect), (3) a single estimate for residual variance and separate estimates for family variance, (4) a separate estimate of residual variance for each cross-type (arbitrarily designated in the SAS code as Type 3 = pure Line 7, Type 4 = pure Line 8, Type 5 = hybrid Line 9 from female 8 × male 7, and Type 6 = hybrid Line 9 from female 7 × male 8) and no variance among families, (5) a separate estimate of residual variance for each type and a single estimate for variance among families, and (6) a separate estimate of residual variance for each type and separate estimates for family variance (see also Garland et al. 2011; Hannon et al. 2011). Analyses from the last, most complex model are presented here.

### Heritability estimates by the animal model

We estimated the heritability of wheel running and its components using an “animal model,” a special type of mixed-effects model, developed in the animal breeding and quantitative genetics literature, that can incorporate the information contained in the pedigree of a population to partition phenotypic variance into different genetic and environmental sources (Wilson et al. 2010). We used the same pedigree for these mice as published previously up to generation 31 (Careau et al. 2013, 2015) to which we added information up to generation 78 (hybrid generation 10). The pedigree included the original 224 mice purchased from Harlan Sprague Dawley, but no information before then (thus, these 224 mice were assumed to be unrelated (Careau et al. 2013, 2015)). Then, we obtained inbreeding coefficients (*F*) using the relationship matrix calculated from the pedigree (Butler et al. 2007). For the parental generation used to create the hybrid line (i.e., generation 68 of the selection experiment), the average (± standard deviation) inbreeding coefficient for HR line 7 was *F* = 0.7087 ± 0.0105 and for HR line 8 was *F* = 0.7198 ± 0.0106.

To estimate heritability of wheel running in the first generation of the hybrid experiment (generation 69 = hybrid generation 1), we subset the pedigree to only the generations relevant to the hybrid line. Thus, the pedigree used to estimate heritability did not include the first 68 generations of selection. Using the animal model in this way effectively assumes that individuals within a line at generation 69 are outbred, which is, of course, untrue (see above). Violating this assumption was necessary to estimate additive genetic variance at generation 69 instead of allowing the animal model to implicitly infer back to the base population of the selection experiment (Careau et al. 2013). To account for inbreeding at the start of the hybrid line, we specified the inbreeding coefficient of all breeders at generation 69 when calculating the A-inverse matrix used in the animal model.

For each trait for which we wanted to estimate heritability, we first standardized the trait to have mean = 0 and standard deviation = 1 separately in each line within each generation. This enabled us to pool generations together and directly compare estimates of variance and regression coefficients between lines. Then, we estimated variance components for each line using linear mixed-effects models, which included fixed effects (age, sex, F coefficient, and wheel freeness) and variance components of common maternal environment (i.e., identity of the mouse’s dam), additive genetic variance (i.e., the identity of the mouse linked with the pedigree), and residual variance. Narrow-sense heritability was calculated as the ratio of the additive genetic variance component divided by the sum of all variance components. We also ran models which included dominance genetic variance (Wolak 2012), but this variance was estimated at the lower boundary of the parameter space in 6 of 9 models and did not change the estimates for additive genetic variance, so we present results from models without this term. Confidence intervals for the variance components were estimated using profile likelihoods with the R package nadiv (Wolak 2012).

We measured cumulative response to directional selection (i.e., selective gain) separately in the sexes as the deviation in each of the 3 lines from the mean of the four C lines, and as the deviation of hybrid line 9 from the average of lines 7 and 8. We also measured the cumulative selection differential in units of standard phenotypic deviation from hybrid generation 1.

### Genetic correlation

We used bivariate “animal models” to estimate the genetic correlation between wheel-running speed and duration. Bivariate animal models included the same fixed and random effects as in the univariate models (see above). By modelling both wheel-running speed and duration as dependent variables in the same model, we were able to estimate the correlation at the common environment, additive genetic, and residual levels using an unstructured general covariance matrix (“us”). Then, we computed the genetic correlation by dividing the additive genetic covariance by the square-root of the product of the additive genetic variances. Traits were standardized to z-scores (mean = 0, SD = 1) separately in each line within each generation. Confidence intervals for the variance components were estimated using profile likelihoods with the R package nadiv (Wolak 2012). Analyses were pooled for both sexes.

## Results

Complete results from the SAS analyses comparing groups (including sample sizes, least squares means, and standard errors) can be found in the Electronic supplementary material Excel file (Supplemental Table S1).

### Parental effects in the F_1_ and grand-parental effects in the F_2_

**Fig. 2:**
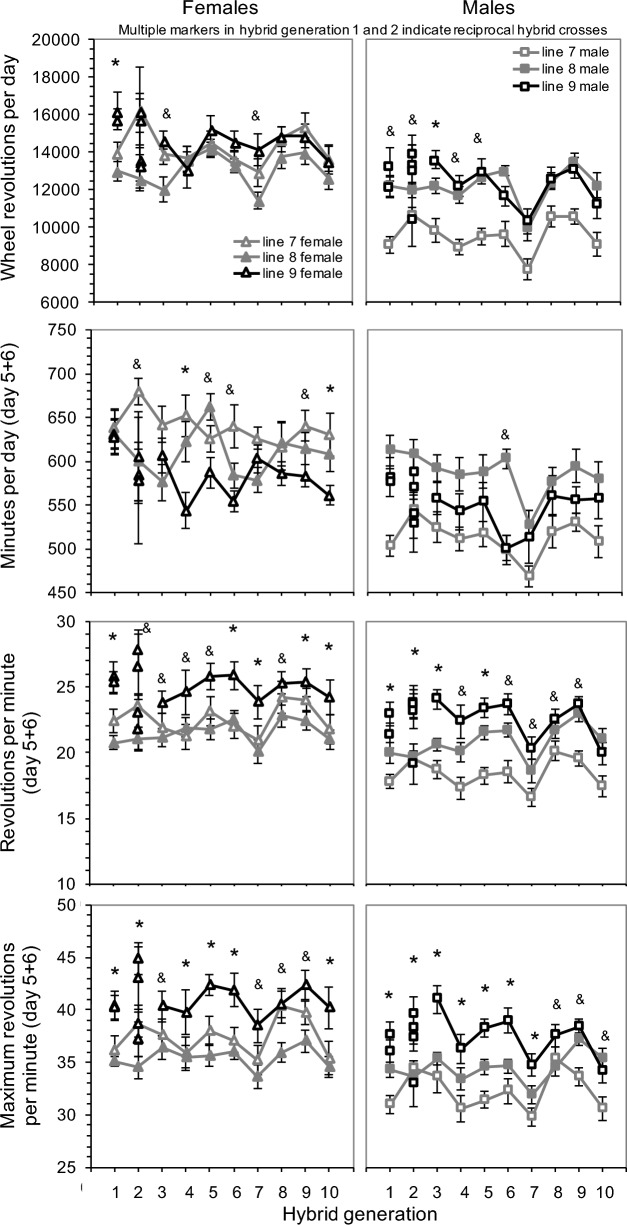
Wheel-running activity for generations 1 (corresponding to generation 69 of the ongoing selection experiment) through 10 of the present experiment, measured as days 5 and 6 of a 6-day exposure to wheels attached to standard housing cages. Parental selected lines are in grey (HR 7 open, HR 8 filled) and the hybrid line is in black, beginning with the F1 animals. In the F1 generation (generation 1), 2 markers are used to indicate the reciprocal hybrid crosses. In the F2 generation (generation 2) 4 markers are used to indicate the 2-way crosses of the reciprocal hybrids (factorial breeding design). Selection was applied to the hybrid line 9 for a total of 9 generations, while also continuing selection in the parental lines 7 and 8. Asterisks (*) indicate when hybrid line 9 was significantly different (*p* < 0.05) from line 7, line 8, and the average of lines 7 + 8. Ampersand (&) symbols indicate when hybrid line 9 was significantly different from one parental line and the average of lines 7 + 8. Values are least-squares means ± standard errors from analysis of covariance models in SAS Procedure Mixed, performed separately for the two sexes (see Materials and Methods), one generation at a time. (Note that the values for generation 1 are the same as in left panels from Fig. 1 and values for generation 2 are the same as in right panels from Fig. 1.)

In the first generation of the hybrid line (F_1_), no parent-of-origin effect was apparent for wheel-running distance, duration, or speed (Fig. 1: left panels; Table S1). That is, the reciprocal crosses were not different from each other (Fig. 2).

The F_2_ revealed interesting grand-parent-of-origin effects. In particular, F_2_ females had significantly increased speed (df = 1,36, F = 6.46, *p* = 0.0155) when the mother was from the F_1_ cross 8F × 7 M compared with F_2_ females with mothers from the F_1_ cross 7F × 8 M (Fig. 1: Revolutions per minute, right panel)*.* In addition, F_2_ males whose mothers were the F_1_ cross 8F × 7 M and whose fathers were the F_1_ cross 7F × 8 M tended to have reduced running distance (*p* = 0.0580) and had significantly reduced running speed (*p* = 0.0121) compared with F_2_ males from the other three F_2_ crosses (Fig. 1: right panels).

**Fig. 3:**
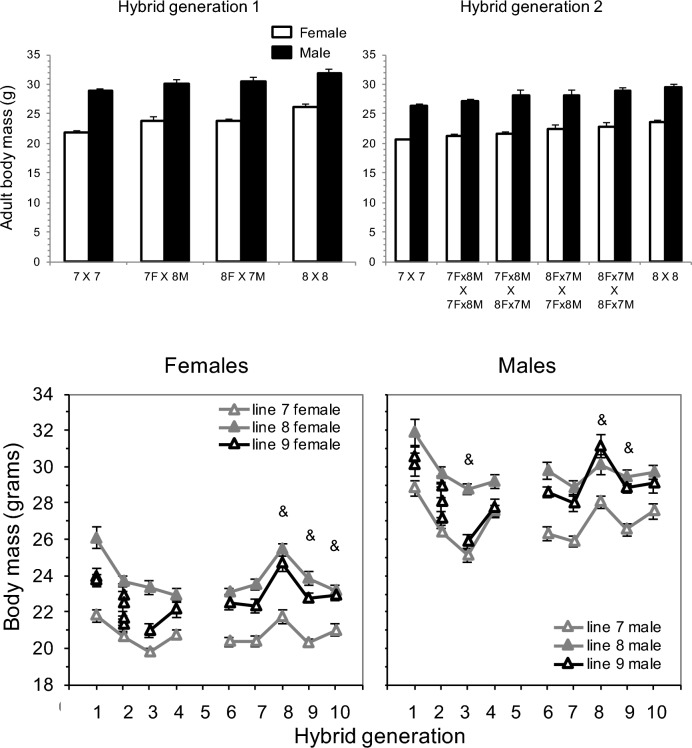
Body mass at the start of wheel exposure. Values are least-squares means ± standard errors from analysis of covariance models in SAS Procedure Mixed, performed separately for the two sexes. Top left) Mice from hybrid generation 1, showing purebred mice and reciprocal hybrid crosses. Top right) Mice from hybrid generation 2, showing purebred mice and the 4-way crosses of the reciprocal hybrid mice. Bottom panels) Body mass, separated by sex, for hybrid generations 1 through 10. Note that values for generation 1 are the same as in top left panel of this figure and values for generation 2 are the same as in top right panel. Body mass is missing for generation 5 due to a broken balance. Parental lines are in grey (HR 7 open, HR 8 filled) and the hybrid line is in black. Ampersand (&) symbols indicate when hybrid line 9 was significantly different from one parental line and the average of lines 7 + 8

**Fig. 4:**
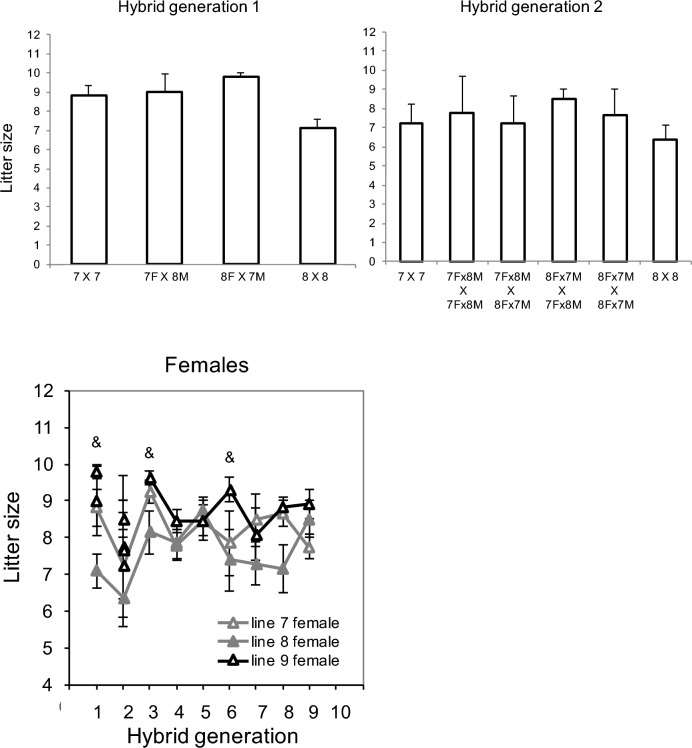
Litter size. Values are least-squares means ± standard errors by line from analysis of covariance models in SAS Procedure Mixed, performed for dams (Note, dams and sires were only paired once, so these numbers are the same for sires). Top left) Mice from hybrid generation 1, showing purebred mice and reciprocal hybrid crosses. Top right) Mice from hybrid generation 2, showing purebred mice and the 4-way crosses of the reciprocal hybrid mice. Bottom panel) Litter size for hybrid generations 1 through 9. Note that values for generation 1 are the same as in top left panel of this figure and values for generation 2 are the same as in top right panel. Parental lines are in grey (HR 7 open, HR 8 filled) and the hybrid line is in black. Ampersand (&) symbols indicate when hybrid line 9 was significantly different from one parental line and the average of lines 7 + 8

Body mass had no apparent parent-of-origin or grand-parent-of-origin effect (Fig. 3; contrasts by cross-type had p > 0.05). All reciprocal groups were intermediate to the two purebred HR lines. These effects were also not significant for litter size (Fig. 4; contrasts by cross-type had p > 0.05).

### Total wheel running

In the F_1_ generation (generation 69), the hybrid line (average of the two reciprocal crosses) had significantly increased total wheel revolutions per day compared to the average of lines 7 and 8, for both females (df = 1,38, F = 12.08, *p* = 0.0013) and males (df = 1,40, F = 10.40, *p* = 0.0025) (Figs. 1, 2; Table S1). In females, the hybrid line was also significantly different from each parental line, but in males, the hybrid line was only statistically different from parental HR line 7, not line 8 (Table S1).

In successive generations, wheel running in the hybrid line generally declined to that of the parental lines (Fig. 2). In females, the hybrid line ran significantly more revolutions/day compared to the average of the parental lines in generations 3 and 7 (Table S1). In males, the hybrid line ran statistically more revolutions than the average of the parental lines for the first 5 generations. In generation 3, the hybrid line was also significantly different from (higher than) the average of the two parental lines (df = 1,29, F = 8.37, *p* = 0.0072). However, from generation 6 on, the males of the hybrid line did not differ in wheel running from the parental lines.

**Fig. 5:**
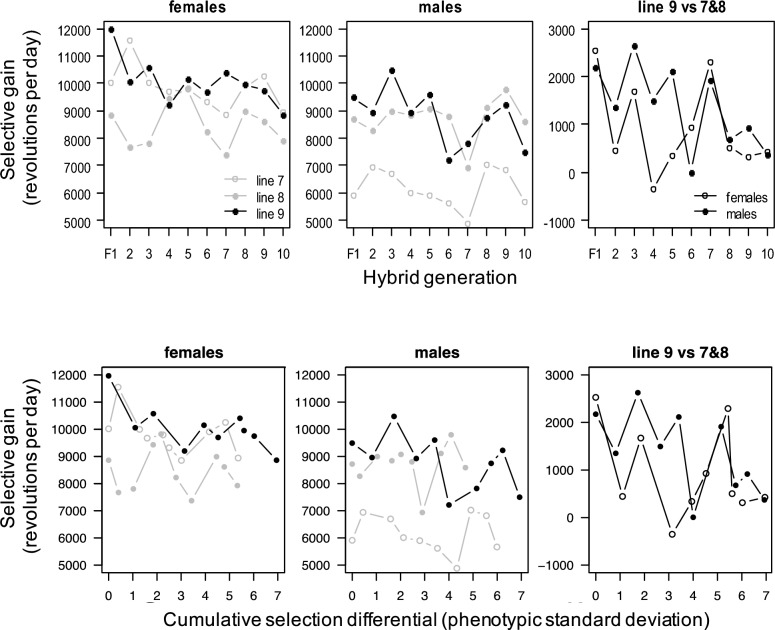
Cumulative response to directional selection (i.e., selective gain) as a function of hybrid generations 1 to 10 (top panels) or the cumulative selection differential (in units of standard phenotypic deviation; bottom panels). Selective gain was measured separately by sex as the deviation of each selected line 7, 8, and 9 from the mean of the four control lines (panels “females” and “males”). Selected gain in the hybrid line 9 was additionally measured as the deviation of line 9 from the average of lines 7 and 8 (panel “line 9 vs 7&8”)

Wheel running shows considerable variation across generations, with all three lines following the same pattern (e.g., dips in generations 7 and 10). This variation is presumed to be caused by intergenerational environmental fluctuations of unknown origin, and likely some amount of apparently endogenous seasonal variation, which is also present in control lines (Careau et al. 2013). One way to control for this variation is to calculate the selective gain by subtracting the average wheel running in control lines from the wheel running in each HR line, which reveal the same pattern either as function of generation (Fig. 5; top panels) or cumulative selection differential (Fig. 5; bottom panels). That is, the hybrid line starts with higher selective gain than lines 7 and 8, but that difference gradually diminishes.

### Duration of wheel running

Wheel-running duration (minutes per day) was measured as the number of 1-min intervals for which the mice showed at least one revolution (Swallow et al. 1998). Generally, the hybrid line was intermediate or lower than parental lines in running duration (Fig. 2, Table S1). Specifically, in females, the hybrid line ran for significantly less time compared to the average of the parental lines in 6 of the 10 generations. In generations 4 and 10, the hybrid line ran significantly fewer minutes per day compared with each parental line. In males, the hybrid line did not differ significantly from the average of the parental lines except in generation 6 (when they ran essentially the same duration per day as HR line 7).

### Average speed of wheel running

Average wheel-running speed (revolutions per minute) was measured as the number of revolutions per day divided by the number of 1-min intervals for which the mice were active per day. Generally, the hybrid line ran at higher speeds than the parental lines (Fig. 2, Table S1). Specifically, in females, the hybrid line ran at significantly higher average speed for all 10 generations compared to the average of the parental lines. At 5 of those time points (generation 1, 6, 7, 9, and 10), the hybrid line also had significantly higher speeds compared with each parental line. In males, the hybrid line had higher average running speed for the first 9 generations compared with the average of the parental lines, but was intermediate in the 10th generation. For 4 of those generations (1, 2, 3, and 5), the hybrid line had significantly higher speeds compared with each parental line.

### Maximum speed of wheel running

The maximum wheel-running speed (maximum revolutions per minute) was measured as the highest number of revolutions run in any 1-min interval, averaged between day 5 and 6. Following the trend for average speed, the hybrid line had higher maximum running speed compared to the parental lines (Fig. 2, Table S1). In females, the hybrid line had significantly higher maximum speeds for all 10 generations compared to the average of the parental lines. At 6 of those time points (generation 1, 2, 4, 5, 6, and 10), the hybrid line also had significantly higher speeds compared with each parental line. For males, the hybrid line also had significantly higher maximum speed for all 10 generations. For the first 7 generations, the hybrid line had significantly higher speeds than each parental line.

### Body mass

Adult body mass (measured before wheel access) of the hybrid mice was intermediate to the parental lines for most generations in both females and males (Fig. 3, Table S1). Specifically, in females, the hybrid line did not differ significantly in body mass compared to the average of the parental lines, except in the last 3 generations. For the last 3 generations of renewed selection, the hybrid line had higher body mass than the average of lines 7 and 8, but only differed significantly from line 7.

In males, the hybrid line did not differ significantly in body mass compared to the average of the parental lines, except in 3 generations. In generation 3, the hybrid line had lower body mass than the average of lines 7 and 8, but only differed significantly from line 8. In generations 8 and 9, the hybrid line had higher body mass compared to the average of the parental lines, but only differed significantly from line 7.

### Litter size

In general, litter size of the hybrid line was slightly higher or intermediate to the parental lines (Fig. 4, Table S1). Specifically, the hybrid line had larger litter sizes compared to the mean of parental lines in generations 1 (df = 1,27, F = 6.06, *p* = 0.0205), 3 (df = 1,19, F = 5.95, *p* = 0.0247), and 6 (df = 1,22, F = 6.05, *p* = 0.0223). (Litter sizes were not recorded for the hybrid line in generation 10.)

### Heritability estimates

Considering data and pedigree information for generations 69 to 78, total daily wheel running was not significantly heritable for either parental line or for the hybrid line, except in females of HR line 7 (Table 1).

**Table 1:** Narrow-sense heritability estimates ± standard errors from analyses of variance components using a pedigree-based linear mixed-model over 10 generations of the hybrid generations (eight generations for the hybrid line due to apparent equipment malfunctions), with number of individuals in parentheses

	HR 7 g0-10	HR 8 g0-10	HR 7 F1-10	HR 8 F1-10	HR 9 F3-10
Total wheel running					
Both sexes	0.03 ± 0.04 (1137)	0.07 ± 0.04 (1156)	0.02 ± 0.02 (863)	0.00 ± 0.02 (777)	0.06 ± 0.05 (691)
Females	0.07 ± 0.07 (564)	0.08 ± 0.07 (564)	**0.22 ± 0.06** (420)	Boundary (386)	0.10 ± 0.08 (342)
Males	0.03 ± 0.06 (573)	0.10 ± 0.06 (592)	Boundary (443)	0.09 ± 0.05 (391)	0.14 ± 0.09 (349)
Duration					
Both sexes	**0.16 ± 0.06**	**0.09 ± 0.04**	0.00 ± 0.02 (863)	0.05 ± 0.04 (777)	**0.15 ± 0.06** (691)
Females	**0.39 ± 0.09**	0.14 ± 0.08	**0.15 ± 0.07** (420)	0.02 ± 0.05 (386)	0.01 ± 0.07 (342)
Males	0.16 ± 0.08	0.10 ± 0.07	Boundary (443)	**0.17 ± 0.07** (391)	**0.28 ± 0.10** (349)
Speed					
Both sexes	0.06 ± 0.05	**0.13 ± 0.04**	0.07 ± 0.03 (863)	0.07 ± 0.05 (777)	0.06 ± 0.05 (691)
Females	0.05 ± 0.07	0.13 ± 0.07	**0.14 ± 0.05** (420)	0.03 ± 0.05 (386)	0.08 ± 0.08 (342)
Males	0.11 ± 0.08	**0.15 ± 0.06**	0.01 ± 0.04 (443)	**0.19 ± 0.06** (391)	0.05 ± 0.07 (349)
Adult body mass					
Both sexes	0.11 ± 0.10	**0.51 ± 0.07**	**0.15 ± 0.06** (863)	**0.24 ± 0.06** (777)	**0.21 ± 0.08** (691)
Females	0.00 ± 0.09	0.16 ± 0.12	0.07 ± 0.08 (420)	0.13 ± 0.09 (386)	**0.35 ± 0.11** (342)
Males	0.11 ± 0.13	**0.61 ± 0.08**	0.04 ± 0.07 (443)	Boundary (391)	0.18 ± 0.11 (349)

Boundary = Unable to be estimated because the additive variance component was getting pushed to be negative to fit the model, but are constrained to be positive

Data were analyzed in the “animal model” with fixed effects (age, sex, measurement batch, F coefficient, and wheel freeness) and variance components of common maternal environment (i.e., identity of the mouse’s dam), additive genetic variance (i.e., the identity of the mouse linked with the pedigree), and residual variance. The “animal model” makes inference back to the starting population, so the pedigree was cut to only include hybrid generation 1. To correct for known relatedness between individuals at hybrid generation 1, each individual in that generation was given the known starting inbreeding coefficient (*F*) according to analysis of the entire pedigree. Traits were standardized to z-scores (mean = 0, SD = 1) separately in each line within each generation. Narrow-sense heritability was calculated as the ratio of the additive genetic variance component divided by the sum of all variance components. Analyses were done separately by sex or pooled for both sexes. Total number of individuals used in the analyses are shown in parentheses. In bold: Estimate is greater than zero and the 95% confidence interval (estimate ± 2 × standard error) excludes zero

The two components of wheel running, duration and average speed, showed a more complicated pattern. Wheel-running duration was heritable for HR line 9 (Table 1 except females), line 7 females, and line 8 males (Table 1). Average wheel-running speed was heritable for HR line 7 females and line 8 males (Table 1).

Adult body mass prior to wheel testing was heritable for HR line 9 (Table 1). When sexes were pooled, body mass was also heritable for line 7 and 8 (Table 1).

### Genetic correlation

Wheel-running duration and speed had a significant negative genetic correlation in HR line 8 and hybrid line 9, estimated by use of animal models (Table 2). For HR line 7, the genetic correlation could not be estimated with certainty due to low genetic variance for duration of running (Tables 1, 2). Estimates of genetic correlation in the base population were positive but not significantly different from zero (by chi^2^ test; Table 2).

**Table 2:** Genetic correlation between speed and duration from animal model analyses

Estimate of genetic correlation using unstructured general covariance matrix models (“us”)			
	*r*_A_	se	Chi^2^ p-value
Base population	0.4905	0.5535	0.4390
Line 7 (F_1_ to F_10_)	− 0.9405^a^	0.8571	0.3612
Line 8 (F_1_ to F_10_)	− **0.8619**	**0.4702**	**0.0310**
Line 9 (F_1_ to F_10_)	− **0.5774**	**0.2628**	**0.0113**
Line 9 (F_3_ to F_10_)	− 0.5653	0.3577	0.0992

Bold indicates Estimate differs from zero based on chi^2^ test

^a^Low genetic variance gives uncertainty to these estimates

Genetic correlations (*r*_A_) between speed and duration were positive in the base population and significantly negative in line 9 (significance from chi-square tests). Estimates for line 7 were unreliable due to low genetic variance. For line 8, the estimate for genetic correlation was significantly negative. Genetic correlations between speed and duration were analyzed in the “animal model” with fixed effects (age, sex, measurement batch, *F* coefficient, and wheel freeness) and variance components of common maternal environment (i.e., identity of the mouse’s dam), additive genetic variance (i.e., the identity of the mouse linked with the pedigree), and residual variance. We used unstructured general covariance matrix models (us). The “animal model” makes inference back to the starting population, so the pedigree was cut to only include hybrid generation 1. To correct for known relatedness between individuals at hybrid generation 1, each individual in that generation was given the known starting inbreeding coefficient (*F*) according to analysis of the entire pedigree. Traits were standardized to z-scores (mean = 0, SD = 1) separately in each line within each generation. Analyses were pooled for both sexes

## Discussion

Here, we attempted to break a selection limit reached during long-term breeding for high voluntary wheel-running behavior in mice. We also tested for changes in the genetic correlation (see also Careau et al. 2015) between the two measured components of daily wheel-running distance, i.e., average speed and duration. After crossing two of the four replicate High Runner (HR) lines, heterosis for wheel-running distance was confirmed in the hybrid F_1_ for both sexes. However, even with nine subsequent generations of directional selection, the hybrid line did not break the prevailing limit in the parental lines. Moreover, the genetic correlation between speed and duration of running evolved from positive to negative in the HR lines, and remained negative in the hybrid line, potentially explaining the persistence of the selection limit. We acknowledge that interpretation of results must be done with caution because we were only able to maintain one hybrid line.

### Genetic variances and covariances of wheel-running distance and its components

Heritability for wheel running was mostly depleted in HR lines 7 and 8 by the start of the hybrid experiment (Table 1). Low heritabilities were not unexpected because these lines had undergone 68 generations of directional selection prior to creation of the hybrid line, although they had maintained heritability at least up until generation ~20 (Careau et al. 2013). [Bult and Lynch (2000) had also estimated non-zero heritabilities for nest building in their selected lines at selection limits.]

Contrary to our prediction, estimates from animal model analyses indicated that heritability for wheel-running distance was not increased in the hybrid line, as compared with the two parental lines, and the estimate did not differ statistically from zero for either sex (Table 1). Hence, little or no response to selection would be expected, and indeed the hybrid line did not respond to selection to any measurable degree (Fig. 2).

Despite the lack of heritability in wheel running, heritability was actually increased in the hybrid line for wheel-running duration (Table 1). Increased heritability in one or both of the components of running distance but not for distance itself can be explained by the presence of a negative genetic correlation between the two components. Indeed, our analyses show that wheel-running duration and speed were positively genetically correlated in the base population, but this correlation had evolved to be negative in the generations used in the present experiment, and remained so in the hybrid line (Table 2). The evolution of a negative genetic correlation between the components of wheel running, where wheel running is a direct predictor of Darwinian fitness under the artificial selection regimen, is analogous to the expected evolution of negative genetic correlations (i.e., trade-offs: Garland et al. 2022) between major components of fitness (e.g., age at first reproduction, fecundity) (Falconer 1981, p. 300; Roff and Fairbairn 2007; Bell 2008, p. 172).

The observed negative genetic correlation could be caused by linkage disequilibrium or pleiotropy of alleles with opposite effects for the component traits. In both cases, alleles with positive effects for running duration and negative effects for running speed (or vice versa) would tend to be inherited together, and selection applied to their product (running distance) should make little or no progress (e.g., Falconer 1981 p. 300; Roff and Fairbairn 2007).

### Heterosis in early generations indicates different genetic architecture for running speed vs. duration

The heterosis observed for wheel running was caused by heterosis for average running speed, but not running duration (Fig. 1, 2). This pattern was also observed for the previous F_1_ cross, but only for males (Hannon et al. 2011) (see also Nehrenberg et al. 2009 for a similar result in a cross of HR line 8 with inbred C57BL/6 J). Previous QTL analyses with an advanced intercross population of mice generated from HR line 8 and C57BL/6 J mice revealed that running speed and duration were affected by entirely different loci (Kelly et al. 2010b). [Other studies of mice have reported co-localized QTL for running speed and duration, but they used a cross of two inbred strains (C57BL/6 J and C3H/HeJ) and measured wheel running over 21 days instead of 6 days (Lightfoot et al. 2008; Leamy et al. 2008).]

As outlined in the Introduction, increased running speed in the hybrid line suggests that the parental lines had some number of alleles lost by genetic drift that were conducive to high running speed, but the two parental HR lines lost different favorable alleles. Thus, the hybrid line inherited “lost” alleles that facilitate higher running speed from both parental lines. Alternatively, the inbreeding (Supplemental Fig. S2) that has unavoidably occurred in these small populations should have resulted in an increased frequency of deleterious recessive alleles present in the homozygous condition at various loci (Charlesworth and Willis 2009), with different loci being affected in each replicate selected line, such that a cross of two replicate lines will result in F_1_ offspring with fewer loci having those deleterious recessives as homozygotes.

Running duration was intermediate in the hybrid line, or even lower in some generations in females (Fig. 2). The observed depression in running duration suggests that separation of beneficial allele combinations via recombination (termed hybrid breakdown) and/or Dobzhansky-Muller incompatibilities were generated (termed outbreeding depression) (Charlesworth and Willis 2009).

### Parental and grand-parental effects

Analyses of parental effects on wheel running (total, duration, average speed, and maximum speed) demonstrated no parent-of-origin effects in the F_1_ generation. That is, the reciprocal F_1_ hybrids showed no statistical difference from one another. Previous research reported parent-of-origin effects in a reciprocal cross between HR line 8 and a control line (Hannon 2010) and in an intercross population between HR line 8 and inbred C57BL/6 J (Kelly et al. 2010a). This discrepancy may be attributable to the fact that mice from HR line 8 were much more similar to those from HR line 7 than they are to Control or C57BL/6 J mice.

In the F_2_ population, however, we found differences between reciprocal hybrids for total wheel running, speed, and maximum speed. These grand-parental effects were further mediated by sex. Specifically, F_2_ female mice whose mothers were 7F × 8 M hybrids had lower total wheel running and speed than mice whose mothers were 8F × 7 M hybrids (although these mothers themselves were did not show any differences in the F_1_), and this was true regardless of the father’s cross-type. Reciprocal crosses of male F_2_ hybrids were not different, expect for one specific cross-type (maternal 8F × 7 M × paternal 7F × 8 M) which had reduced total wheel running and speed. The mechanism for grand-parental effects in the absence of parent-of-origin effects is unclear and beyond the scope of the current study. Some potential mechanisms to explain the sex differences in grand-parental effects (i.e., female vs. male grand-offspring of the same cross-type) is that the allelic combinations (or regulating mechanisms of these combinations), might be found on the X chromosome, mitochondrial DNA, or modulated by epigenetic mechanisms. Discussion of these sex-dependent mechanisms can be found elsewhere (Kelly et al. 2010a).

### Sex differences

As mentioned above, wheel-running duration, which did not exhibit heterosis, was differentially affected in female vs. male hybrid mice. In addition, sex-specific effects were observed in the heritability estimates, further indicating the different underlying genetic architecture of wheel running and its component traits between the two sexes. One potential interpretation is that some alleles that affect running duration may be connected to sex chromosomes. Counter to this hypothesis, no sex-specific QTL were found for wheel running or component traits in a study of an advanced intercross population of HR line 8 and C57BL/6 J mice (Kelly et al. 2010b). However, interpretation is limited because they used just 30 markers on the X chromosome and no markers on the Y chromosome (Kelly et al. 2010b). Another study utilizing the same intercross at a later generation reported 10 QTL for exercise across 20 chromosomes (including the X chromosome), but none of these interacted with sex (Leamy et al. 2012).

Other potential mechanisms of sex differences (mitochondrial DNA, epigenetics, or environmental effects) are discussed elsewhere (Kelly et al. 2010a, b). Identifying the specific mechanism of the observed sex-specific heterosis is outside the scope of the current study, but future analyses of the genetic samples of these mice will yield insight into potential mechanisms of sex differences.

### Body mass

In the first few generations, the hybrid line had intermediate values of body mass compared with HR line 7 and 8, indicating additive inheritance, as has often been reported for mouse body mass (e.g., Falconer 1955). Crosses of HR line 8 with inbred C57BL/6 J (Nehrenberg et al. 2009) and of wild house mice and ICR mice (Dohm et al. 1994) also reported intermediate values for body mass in F_1_ hybrids. After the first few generations, however, the hybrid line became more similar to HR line 8, implying net dominance of the alleles found in HR line 8 for body mass. Heritability was found for body mass in HR lines 7 and 8 (pooled sexes), and in hybrid line 9 (pooled sexes and females; Table 1).

## Comparison with Bult and Lynch (2000)

The current study was inspired by the hybrid cross experiments of Bult and Lynch (2000). Similarities included long-term selection for a behavioral trait, similar population sizes, replicated selected lines, use of within-family selection, selection limits being reached at generation ~20, and genetic variance being maintained at the limit. At generation 46, they created hybrid lines by crossing their selected lines and were able to break the selection limits after 10 generations of renewed selection (Bult and Lynch 2000). Despite the similarities in these studies, our hybrid line did not break the selection limit. Aside from the obvious difference between these studies in the behavior under selection (thermoregulatory nest building vs. wheel running) and direction of selection (high and low vs. only high-selected lines), other discrepancies may have contributed to the difference in outcome, such as the use of different base populations (e.g., see Zombeck et al. 2011).

Perhaps most importantly, Bult and Lynch (2000) allowed random mating in the first 3 generations of the hybrid line before renewing selection for 10 more generations. In our experiment, we opted to select on the hybrid line from hybrid generation 1. This was partly due to limitations based on the number of mice we could keep for this experiment while maintaining the other selected and control lines of the selection experiment. Although we did not have random mating, the factorial design in creating the F_2_ allowed some allele mixing.

## Concluding remarks

Our results suggest that the genetic architecture (specifically, the negative genetic correlation between wheel-running component traits duration and speed) constrained the hybrid line from increasing voluntary wheel running beyond the selection limit experienced by the parental lines. Even with renewed genetic variation for duration of wheel running, hybrids were not able to break the selection limit on total wheel running, because hybrid vigor was countered by one or more forms of hybrid depression. That is, the two benefits of a hybrid line (1. reduction of slightly deleterious homozygous alleles found in parental lines after generations of inbreeding, and 2. new, beneficial combinations of genes) may have been outweighed by breaking up good combinations (i.e., favored by past selection) that were already in each parental line or by the creation of new, harmful combinations of alleles. Aside from the issue of hybrid vigor versus depression, the possible contributions of dominance, overdominance or pseudo-overdominance to the observed heterosis for wheel running in the first few generations are unknown. Samples of breeders from all 10 generations of the hybrid line have been preserved for future genomic analyses, which may uncover these genetic mechanisms.

## Supplementary Information

Below is the link to the electronic supplementary material.

Supplementary file1 (DOCX 103 KB)

Supplementary file2 (XLSX 319 KB)

## Data Availability

Code and data for performing all analyses will be available from the corresponding author on request.
