# Supplementary material for: Can a Hybrid Line Break a Selection Limit on Behavioral Evolution in Mice?
Source: Behav Genet. 2024 Dec 5;55(1):43–58. doi: 10.1007/s10519-024-10209-7 (PMC11790750; doi:10.1007/s10519-024-10209-7)
Supplement: Supplementary file 1 — Supplementary file1 (DOCX 103 KB) [file 10519_2024_10209_MOESM1_ESM.docx]

# Supplemental Material

Table S1. Excel file with full statistical results from SAS Procedure Mixed comparing groups for each trait by generation (Hybrid_SAS_Results_SV_16_UPLOAD.xlsx).

Fig. S1. Experimental timeline. See Materials and Methods for further explanaton.


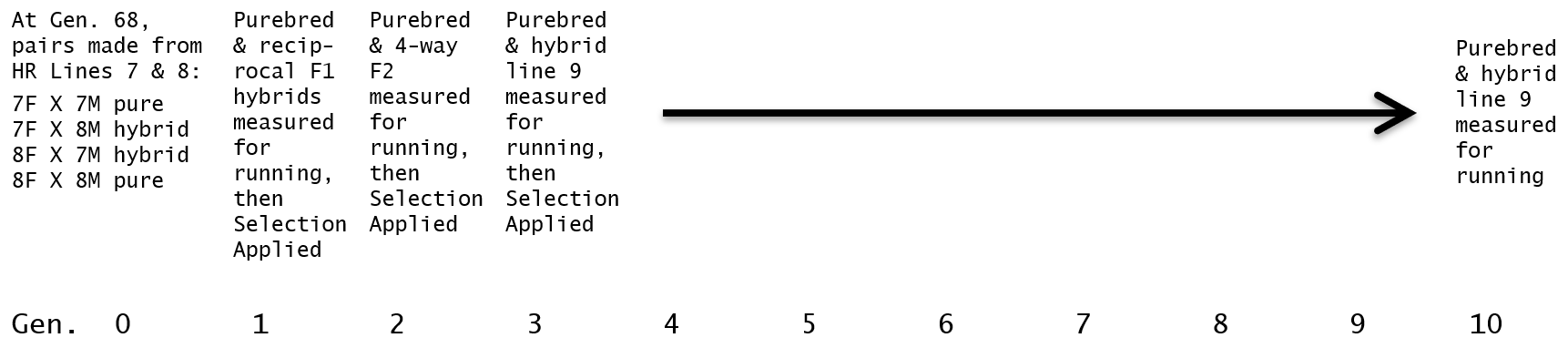


Fig. S2. To obtain the inbreeding coefficient, we calculated the relationship matrix from the pedigree of these individuals (Butler et al. 2007). We used the same pedigree for these mice as published previously up to generation 31 (Careau et al. 2013, 2015) to which we added information up to generation 78 (Dewan et al. 2017). The pedigree included data from the original 224 mice purchased from Harlan Sprague-Dawley, but no information before then (thus, these 224 mice were assumed to be unrelated (Careau et al. 2013, 2015)). As expected, the F-coefficient of the hybrid F_1_ generation was 0, as they were offspring of two distantly related populations. However, from generation 2 of hybrid line 9, *F* increased to half the *F* of the parental lines. Every generation, *F* increased slightly, but did not reached *F*=0.5 for the hybrid line over 10 generations.
